# Does internet use improve employment?——Empirical evidence from China

**DOI:** 10.1371/journal.pone.0301465

**Published:** 2024-04-16

**Authors:** Yunqiu Zhan, Shuwen Yang

**Affiliations:** 1 School of Marxism, Chengdu Technological University, Chengdu, Sichuan Province, China; 2 Research Institute of Social Development, Southwestern University of Finance and Economics, Chengdu, Sichuan Province, China; Public Library of Science, UNITED STATES

## Abstract

Achieving comprehensive and high-quality employment is essential to achieving new levels of people’s well-being. The advancement of Internet technology not only affect the massiveness of employment, but also the quality of that. On the basis of constructing an employment quality evaluation index system, this article uses CLDS (China Labor-force Dynamics Survey) data to explore the impact of Internet use on the employment quality of workers and its underlying mechanisms. The results reveal that Internet use has a significant positive impact on improving the employment quality of workers. As the quantile of employment quality increases, internet use has a greater impact on workers with a lower employment quality quantile. In addition, the use of Internet has a more significant promoting effect on the employment quality of rural and female workers. From the perspective of mechanism, Internet use can increase workers’ social capital and influence their employment quality through the accumulation of social capital. Based on this, countermeasures and suggestions are put forward from the aspects of increasing investment and construction of Internet infrastructure, further perfecting the reform of household registration system, promoting human capital investment and social capital construction.

## 1 Introduction

With the advent of the digital age, the digital technology revolution represented by the Internet is driving the modernisation of traditional modes of production and lifestyles. Compared with traditional communication media, the Internet has network-specific attributes such as inter-temporal capability, as well as strong spillover and externality. The Internet has raised the level of informatisation, driven a new round of global scientific and technological revolution, and had an impact on various socio-economic fields. From the perspective of the employment field, the Internet has created new jobs for workers while promoting industrial restructuring. According to the White Paper on China’s Digital Economy Development and Employment (2021), there were 256 million jobs in China’s digital economy in 2020, up 15 percent year-on-year. "Achieving higher-quality employment" is a strategic choice for China’s current economic development, and the Internet will not only affect the quantity of employment, but also the quality of employment. So, how will Internet technology affect employment quality while also affecting employment quantity? What is the internal mechanism of Internet affecting employment quality? It is of great significance to analyze these questions against the double background of rapid development of digital technology and advocacy of high-quality employment.

Internet-related studies were quite rich. The existing literature focuses on the following areas: First is the impact of the Internet on household economic well-being. Studies have shown that Internet use can reduce farmers’ costs of accessing information and enable them to make better production decisions, which can help increase farmers’ income and expenditures [1, 2], but it may widen the income gap between employers and employees as well [3]. In addition, Internet use can also reduce the incidence of poverty and increase the diversity of consumption of the population [4, 5]. Second is the impact of Internet use on agricultural productivity and economic efficiency. Relevant studies have shown that the Internet can increase the rate of agricultural production [6], reduce the inequality of agricultural productivity [7], and increase farm profits [8]. Therefore, the government should improve the construction of rural Internet infrastructure and increase the Internet penetration rate. Third is the impact of Internet use on e-commerce, online banking use and diversity of risky financial assets. In terms of e-commerce, the adoption of e-commerce can significantly increase household incomes [9], while Internet use can help increase the probability of farmers engaging in e-trade [10]. In terms of online banking, scholars suggest that customers’ skills in using the Internet need be improved, and website design should be more visually appealing to increase users’ willingness to use online banking [11–13]. In terms of risky financial diversity, relevant studies have shown that Internet use has an impact on the diversity of risky financial assets, and this impact is more obvious in the female group [14]. Fourth is the impact of Internet use on the sense of happiness and social justice. Internet use is closely related to mental health [15], with one view suggesting that it reduces feelings of loneliness and sadness, thereby increasing subjective well-being [16]. Another viewpoint is that Internet use reduces people’s life satisfaction and happiness [17], as well as the sense of social justice [18].

It can be seen that a large number of studies have explored the impact of Internet use on various socio-economic aspects, but few studies have focused on the relationship between Internet use and employment quality. Even if a few studies have explored the impact of Internet use on the quality of employment, they have mostly analyzed it from a single dimension and lacked a comprehensive perspective. Combined with existing research, the contributions of this article are: first, while a large number of studies have discussed the impact of Internet use on the economic well-being of rural households, crop productivity, farm economic performance, e-commerce, online banking use, happiness, and social equity, few studies have analyzed the impact of Internet use on the quality of employment. The only studies on the impact of the Internet on employment quality mainly analyze the impact of Internet use a certain dimension of employment quality, lacking a comprehensive perspective. This article adopts the method of factor analysis to measure the index of employment quality and comprehensively analyze the impact of Internet use on employment quality. Second, unlike existing studies that mainly analyze employment quality at the macro level, this article uses micro data to analysize employment quality, which not only includes factors such as social security, salary level, labour contract, working hours, etc. at the macro level, but also pays attention to the subjective evaluation of workers on their jobs, such as job satisfaction and job security, so as to provide a more comprehensive measurement of employment quality. Thirdly, in addition to directly affecting the quality of employment, the use of the Internet also indirectly affects the quality of employment through other intrinsic mechanisms, and this article makes the study more in-depth by analyzing the intrinsic mechanisms of the Internet affecting the quality of employment.

The remainder of the article is structured as follows: Section 2 combs the relevant literature and puts forward the theoretical hypothesis. Section 3 introduces the data sources, variables and empirical models. Section 4 reports the results of empirical analysis, including baseline regression, quantile regression, heterogeneity analysis, robustness test and internal mechanism analysis. Section 5 is the discussion. Section 6 offers implications, limitations and future recommendations.

## 2 Literature review and hypothesis

### 2.1 The impact of the Internet on the quality of employment

Research on the impact of the Internet on employment has centered on three aspects: the quantity of employment, the structure of employment, and the quality of employment. In terms of the quantity of employment, there is a controversy about whether the Internet has increased or decreased the number of jobs. One viewpoint is that the Internet reduces jobs. According to Marx’s theory of relative surplus population, the organic composition of capital will cause a decrease in the demand for the labor force population, thus creating a relative surplus population. With the development of the Internet, materialized labor will further replace human labor, and there is a certain extrusion effect on jobs [19, 20]. Another viewpoint is that the Internet will increase jobs, and the digital platform integrates resources and information from all aspects, which can provide more job opportunities for job seekers. Some scholars have examined the impact of the Internet on local employment rates based on panel data on broadband coverage in West Germany, and the results show that the popularization of the Internet can significantly increase employment rates [21]. Other studies have shown that the resources and information provided by Internet platforms can provide job seekers with more employment opportunities and choices [22]. In terms of employment structure, the development of Internet technology has an important impact on the adjustment of employment structure, and most scholars’ studies show that digital technology will increase the employment of high-skilled and low-skilled workers, but reduce the employment of middle-skilled workers, the employment structure in the Internet era will show the characteristic of "bipolarization" [23].

Employment quality is a comprehensive concept, which can roudly reflect the working conditions that workers receive when they engage in social work, such as the level of wages and benefits, the length of working hours, whether labour rights and interests are protected, and the subjective satisfaction of workers with the work they do [24–26]. When scholars discuss the impact of Internet use on employment quality, they mostly analyze from a single dimension and lack a comprehensive perspective. (1) In terms of employment opportunities, the Internet can shorten workers’ job search time, reduce the cost of searching for a job, and increase the probability of getting a job [27, 28]. (2) In terms of labour skills, the Internet technology has enriched people’s material and cultural life and at the same time promoted the improvement of labour skills [29, 30]. (3) In terms of wages, the use of the Internet has a positive effect on raising the wage level of workers, workers who use the Internet can generate a significant wage premium [31]. (4) In terms of work autonomy, workers who work remotely through the Internet can achieve a better work-life balance and increase their work autonomy [32, 33]. (5) In terms of working time, the Internet has a double effect on the working time of workers, as it may increase productivity and reduces working time, it can lengthen the actual working time of workers due to the flexibility of office forms as well [34].

In general, although some studies analyzed the impact of the Internet on employment quality, they analyzed a particular dimension of employment quality or several dimensions separately, lacking a comprehensive perspective and relating mechanisms analysis. This article uses micro data to construct an employment quality index system, analyzing the impact of the Internet on the employment quality composite index and exploring the inner mechanism of the Internet’s impact on employment quality. The main effects of the Internet on employment quality are as follows: The first is the efficiency-enhancing effect. Emerging technologies such as the Internet have brought about new forms of business, which are mainly manifested in the platformisation, flexibilisation and borderlessness of employment. Under the premise that the total amount of work is fixed, workers can improve their work efficiency and reduce their working hours through instant communication and data transmission through the Internet, which improves their job satisfaction. The second is the wage enhancement effect. With the popularity of the Internet, economic development is gradually digitised, and various industries are also continuously extended and expanded due to "Internet+", which has boosted and activated the consumer Internet. Now it is integrating manufacturing and agriculture, promoting the digital transformation of agriculture and industry through the industrial Internet, realising digital agriculture and service-oriented manufacturing, giving rise to new industries and new modes, and creating a large number of new employment modes, thus promoting the continuous optimisation of the employment structure, and providing workers with better career development and salary compensation. The third is the quality enhancement effect. More highly qualified workers are needed in the Internet Age, which places higher demands on workers’ working ability and motivates them to continuously improve their working ability so as to obtain better promotion opportunities. The fourth is the stability enhancement effect. The Internet helps to increase public supervision of enterprises, protect workers’ rights and interests, and improve the security and stability of employ-ment. The fifth is the security-enhancing effect. The Internet can spread legal knowledge and raise workers’ awareness of contracts, as well as protecting workers’ legitimate rights and interests through encouraging enterprises to sign formal employment contracts with workers and taking out social insurance.

Based on this, Hypothesis I is proposed: Internet use can improve the workers’ quality of employment.

### 2.2 Internet use, social capital and quality of employment

The study of social capital theory can be traced back to the early 20th century, when the Hanifan (1916) referred it in The Community Center. In Hanifan’s view, social capital was both a prerequisite for collaboration between members and a resource for strengthening social networks [35]. On this basis, Bourdieu (1991) explained social capital in a more systematic way that social capital enhanced individual interests and was both a collection of potential resources and an institutionalized network of relationships [36]. Lin (2002) argued that social capital was not possessed by individuals and could not be acquired without effort, but needed to be acquired from social networks through practice. Social resources would play a more important role than individual resources at a later stage [37].

The rapid development of the Internet broadened people’s social networks. Social resources are better integrated with the increading accessibility of information. The Internet provids a platform for people to communicate and share information with each other, which has a positive effect on the level of social capital [38]. The Internet can broaden social networks and has a positive impact on the enhancement of social capital [39]. Social capital is also seen as an individual’s ability to mobilize and absorb resources from a wide network. The richer the resources, the more opportunities they have for quality employment. Thus, social capital can improve the quality of employment for workers [40].

Based on this, Hypothesis II is proposed: Internet use can directly improve the workers’ quality of employment while indirectly improve the workers’ quality of employment through the accumulation of social capital.

## 3 Methods

### 3.1 Data source

This article uses data from the CLDS, a survey planned and implemented by Sun Yat-sen University, with the latest survey conducted in 2018 and data officially released in 2020. The CLDS collects data at three levels: individual labor force, household and village residence, with the working-age population as the main study target, covering internet use and variables related to personal characteristics, Human capital characteristics, occupational characteristics, household characteristics and other variables, in line with the aims and themes of this study. The data covers 497 villages in 29 provinces (municipalities and autonomous regions) in China, making it a large, interdisciplinary, dynamic and regular tracking survey that is highly representative. The employed population aged 18–60 still in the labor market are retained in conjunction with the study objectives, yielding a sample of 3,983 people after dealing with missing values.

### 3.2 Variables

#### Internet use

Current indicators on measuring people’s Internet use behavior mainly include: whether they use the Internet, the time they use the Internet, and the frequency of Internet use. In this article, Internet use is measured by how often workers use the Internet, use respondents’ 4-point scale answers to the questions “Do you surf the Internet?” to measure Chinese workers’ Internet use, with 1 indicating “never use”, 2 indicating “rarely use”, 3 indicating “sometimes use” and 4 indicating “often use”.

#### Quality of employment

The measurement of employment quality is relatively complex, including both objective factors such as job income, working hours, social security and labor relations, and subjective factors such as job satisfaction. In this article, we refer to Leschke’s (2014) study and combine the CLDS-related options to construct an employment quality index system 25, using factor analysis to extract eight dimensions of factors (with an explanatory rate of 0.759), calculating the employment quality index and transforming it to obtain an employment quality index variable with values between 0 and 100, as shown in Table 1.

**Table 1:** Employment quality index system.

Index name	Primary index	Secondary index	Factor weight
**Employment quality index system**	**Fator1** **Social security factor**	Endowment insurance	0.266
		Medical insurance	
		Employment injury insurance	
		Maternity insurance	
		Unemployment insurance	
		Housing accumulation fund	
	**Fator2** **Subjective satisfaction factor**	Satisfaction of the relationship between partners	0.207
		Satisfaction with ability display	
		Satisfaction of job respect	
		Satisfaction with the opportunity to express opinions	
	**Fator3** **Work autonomy factor**	Autonomy of task content	0.159
		Autonomy of work schedule	
		Autonomy of work intensity	
	**Fator4** **Work safety factor**	Job safety satisfaction	0.103
		Work environment satisfaction	
	**Factor5** **Career development factor**	Opportunity for advancement	0.073
		Interesting work	
	**Fator6** **Salary compensation factor**	Work income	0.066
	**Fator7** **Labor time factor**	Working hours	0.064
	**Fator8** **Labor relation factor**	Trade union organization	0.062

#### Social capital

There is no consensus in the academic community on the measurement of social capital. Some studies use trust as a proxy variable for social capital [41], others measure social capital by participation in institutional organizations [42]. According to Granovetter’s (1985) theory of embeddedness, economic decision-making processes need to take place in social interactions and economic behavior is embedded in social networks. Some factors in social networks, such as trust, family and friendships, may lead to altruistic economic behavior [43]. Altruistic behavior refers to acts of helping others to gain benefits at the expense of one’s own interests in times of difficulty or need. In this article, based on Granovetter’s theory of embeddedness, the number of people who can ask for help in times of crisis is used as a proxy variable for the mediating variable of social capital. 1 indicating “the number of people who can ask for help is 0”. 2 indicating “the number of people who can ask for help is 1–5”. 3 indicating “the number of people who can ask for help is 6–10”. 4 indicating “the number of people who can ask for help is more than 10”.

#### Instrumental variables and control variables

The instrumental variable is the mean value of the frequency of Internet use in the worker’s community. As personal characteristics, human capital characteristics, occupational characteristics and family factors also affect the quality of employment of individuals, this article also includes age, gender (female = 0, male = 1), household registration (rural = 0, urban = 1), political affiliation (non-party member = 0, party member = 1), health (very unhealthy = 1, relatively unhealthy = 2, average = 3, healthy = 4, very healthy = 5), years of education, vocational certificate (no = 0, yes = 1), Mandarin level, full-time work (no = 0, yes = 1), work in the system (no = 0, yes = 1), size of work unit, family member relationship, and family economic level are used as control variables. The all-sample distribution is shown in Table 2.

**Table 2:** Summary statistics.

Variables	Meaning and measurement	Mean	Min	Max
Quality of employment	Composite index of employment quality (obtained by factor analysis)	57.31	0	100
Internet use	How often workers use the Internet (never = 1, rarely = 2, sometimes = 3, often = 4)	2.49	1	4
Social capital	Number of people to turn to when in trouble (None = 1, 1–5 people = 2, 6–10 people = 3, more than 10 people = 4)	2.62	1	4
Personal characteristics:				
Age	Age of the worker (year)	38.83	18	60
Gender	Gender of the worker (female = 0, male = 1)	0.55	0	1
Household registration	Household registration of the worker (rural = 0, urban = 1)	0.49	0	1
Political identity	Political identity of the worker (non-party member = 0, party member = 1)	0.16	0	1
Human capital characteristics:				
Health	Health level of the worker (very unhealthy = 1, relatively unhealthy = 2, average = 3, healthy = 4, very healthy = 5)	3.93	1	5
Education	Education of the worker (year)	11.51	0	22
Occupational certificate	Whether the worker has an occupational certificate (no = 0, yes = 1)	0.29	0	1
Mandarin level	Mandarin level of the worker (from 1 to 5, with higher scores representing higher levels of Mandarin)	4.13	1	5
Occupational characteristics:				
Full-time work	Whether the worker works full-time (No = 0, Yes = 1)	0.92	0	1
Work in the system	Whether the worker works in the system (no = 0, yes = 1)	0.23	0	1
Size of work unit	Number of workers in the unit (<50 = 1,50–100 = 2, 200–500 = 3, > 500 = 4)	2.07	1	4
Family factors:				
Family member relationship	Family member relationship of the worker (from 1 to 10, with higher scores representing better family member relationships)	7.42	1	10
Family economic level	Family economic level of the worker (from 1 to 10, with higher scores representing better family economic level)	6.10	1	10

### 3.3 Empirical model

Linear regression is a model used to establish the relationship between a dependent variable and one or more independent variables. Simple linear regression involves one independent variable, while multiple linear regression involves multiple independent variables. This model predicts or explains the value of a dependent variable by considering multiple independent variables. Factors influencing employment quality are diverse, so this article constructs a multiple linear regression model of the impact of Internet use on employment quality, and sets the following regression equation:

$${Employ}_{i}=\alpha_{0}+\alpha_{1}{Internet}_{i}+\alpha_{2}X_{i}+\varepsilon_{i}$$ (1)

*Employ*_*i*_ represents every worker’s employment quality. *Internet*_*i*_ stands for Internet usage frequency. *X*_*i*_ stands for other control variables, including age, gender, household registration, political affiliation, health, years of education, vocational certificate, Mandarin level, full-time work, work in the system, size of work unit, family member relationship, and family economic level.

Quantile regression is a regression analysis method used to explore changes in the distribution of the dependent variable under different conditions. Unlike traditional ordinary least squares regression, quantile regression focuses on the conditional distribution of the dependent variable at different quantiles, rather than just the average level of change. In quantile regression, we aim to capture a more comprehensive view of the data features by fitting the conditional distribution at various quantiles. This method is more sensitive to outliers and asymmetry in data distribution. To examine the differential impact of internet usage on the quality of employment across different quantiles, the following regression equation is set:

$$Q_{\theta }\left(\frac{{Employ}_{i}}{Z_{i}}\right)=\varphi^{\theta }Z_{i}$$ (2)

Z_*i*_ in Eq (2) represents the core explanatory variable mentioned in (1), $Q_{\theta }(\frac{{Employ}_{i}}{Z_{i}})$ represents the conditional quantile of *Employ*_*i*_, θ represents the examined quantile, *φ*^*θ*^ is the vector of coefficients corresponding to θ, the estimation method is the minimization of the absolute deviation. The computation method of *φ*^*θ*^ in Eq (2) is shown as in Eq (3):

$$\varphi^{\theta }=argmin\{\sum_{i\;{Employ}_{i}\geq X_{i}\varphi }{\theta /Suratio}_{i}-X_{i}\varphi /+\sum_{i,{Suratio}_{i}<X_{i}\varphi }{(1-\theta )/Suratio}_{i}-X_{i}\varphi /\}$$ (3)

Mediation analysis model is typically utilized to determine whether the impact of an independent variable on a dependent variable is direct or indirectly facilitated through intermediary variables. This analysis aids in obtaining a more comprehensive understanding of the relationships and mechanisms between variables. In examining methods related to testing the mediation mechanism, stepwise regression analysis has been widely employed [44]. Referring to this study, we constructs the following equation based on Eq (1):

$${Capital}_{i}=\beta_{0}+\beta_{1}{Internet}_{i}+\beta_{2}X_{i}+\varepsilon_{i}$$ (4)

$${Employ}_{i}=\gamma_{0}+\gamma_{1}{Internet}_{i}+\gamma_{2}{Capital}_{i}+\gamma_{3}X_{i}+\varepsilon_{i}$$ (5)

To test the mediating effect of *Capital*_*i*_ in *Internet*_*i*_ affecting *Employ*_*i*_, first test the coefficient *α*_1_, if *α*_1_ is not significant, stop the mediation test; if *α*_1_ is significant, test β_1_ and γ_2_. If β_1_ and γ_2_ are significant, then test γ_1_; if γ_1_ is significant, part of the intermediary effect is valid, if γ_1_ is not significant, it is a complete intermediary effect. If β_1_ and γ_2_ at least one is not significant, conduct sobel test. If it passes the test, the intermediary effect exists. And vice versa, the intermediary effect does not exist.

## 4 Empirical results

### 4.1 Basic analysis

Table 3 presents the Baseline results. Model 1 only includes the core explanatory variable Internet use frequency. Model 2-Model 5 adds control variables related to personal characteristics, human capital characteristics, occupational characteristics and family characteristics respectively. With the addition of the control variables, the model’s goodness-of-fit R^2^ increases, indicating that the model’s explanatory strength gradually increases and the variables are reasonably selected. From model 5, it can be seen that for every 1 unit increasing in the frequency of Internet use, the quality of employment of workers increases by 0.518. Internet use have a positive effect on the quality of employment of workers, which verifies the Hypothesis I.

**Table 3:** Baseline results.

	Model 1	Model 2	Model 3	Model 4	Model 5
	Quality of employment	Quality of employment	Quality of employment	Quality of employment	Quality of employment
Core explanatory variables:					
Internet usage frequency	2.032***	1.267***	0.524***	0.585***	0.518***
	(0.110)	(0.122)	(0.133)	(0.131)	(0.131)
Personal characteristics:					
Age		0.335***	0.359***	0.253***	0.221**
		(0.091)	(0.089)	(0.088)	(0.088)
Square of age		-0.004***	-0.003***	-0.002**	-0.002**
		(0.001)	(0.001)	(0.001)	(0.001)
Male		0.494*	0.475*	0.583**	0.531*
		(0.282)	(0.278)	(0.274)	(0.274)
Urban resident registration		4.441***	2.748***	2.052***	2.037***
		(0.308)	(0.335)	(0.339)	(0.338)
Party member		2.235***	1.108***	1.017***	0.851**
		(0.376)	(0.382)	(0.376)	(0.377)
Characteristics of human capital:					
Health condition			1.007***	0.989***	0.909***
			(0.170)	(0.167)	(0.168)
Years of education			0.466***	0.398***	0.362***
			(0.056)	(0.055)	(0.056)
Professional certificate			1.614***	1.150***	1.037***
			(0.316)	(0.316)	(0.316)
Mandarin level			0.799***	0.743***	0.726***
			(0.170)	(0.168)	(0.168)
Occupational characteristics:					
Full-time				2.056***	2.109***
				(0.557)	(0.555)
Within the system				2.674***	2.660***
				(0.338)	(0.337)
Unit scale				0.632***	0.586***
				(0.107)	(0.108)
Family characteristics:					
Family member relationship					0.191**
					(0.094)
Family economic situation					0.219**
					(0.096)
Observations	3,983	3,983	3,983	3,983	3,983

Notes: ①Robust standard error in parentheses.

②*** p<0.01

** p<0.05

* p<0.1.

From the perspective of individual demographic characteristics, the quality of employment shows an inverted U-shaped relationship with age. As age increases, the quality of employment initially rises and then declines. After adding control variables, male employment quality is 0.531 higher than female, indicating the prevalence of gender stratification in the employment sector where females generally occupy a disadvantaged position in the labor market. Urban registered residents’ employment quality surpasses rural residents by 2.037, constrained by the urban-rural dual household registration system, leading to higher employment quality for urban registered residents. Moreover, compared to non-party members, party members exhibit a 0.851 higher employment quality.

From the perspective of human capital characteristics, full-time employees within state-owned enterprises exhibit higher employment quality. Employment quality also correlates positively with organizational scale, with a 0.586 increase in employment quality for every unit increase in organizational scale. Examining family characteristics, better family relationships and improved family economic status correlate with higher employment quality.

From the perspective of occupational features, after incorporating all control variables, full-time workers experience a 2.109 increase in employment quality, while those within state-owned enterprises witness a 2.660 increase in employment quality. Employment quality also correlates positively with organizational scale, with a 0.586 increase in employment quality for every unit increase in organizational scale.

From the perspective of family characteristics, after incorporating all control variables, better family relationships and improved family economic status correlate with higher employment quality.

### 4.2 Quantile analysis

Table 4 presents the quantile regression results from the 10th percentile to the 90th percentile respectively, indicating that Internet use has a significant positive impact on employment quality at all quantiles. But there are certain differences in the variation of coefficient values at different quantiles. Specifically, with the continuous improvement of the quantile of employment quality, the regression coefficient shows a downward trend, from 0.761 at the 10th quantile to 0.595 at the 25th quantile, 0.557 at the 50th quantile, 0.458 at the 75th quantile and 0.379 at the 90th quantile, indicating that Internet use has a greater impact on workers with lower quantile of employment quality. The possible reason for this phenomenon is that the positive effect of Internet use on employment quality has the possibility of diminishing marginal utility. With the improvement of Internet use, Internet has gradually become an vital part of people’s work and life. Compared to workers with higher employment quality, those with lower employment quality have weaker job competitiveness. Accordingly, Internet use will also be more sensitive to the effects of the later ones.

**Table 4:** Quantile regression results.

	Model 1	Model 2	Model 3	Model 4	Model 5
	Quality of employment	Quality of employment	Quality of employment	Quality of employment	Quality of employment
Quantile level	0.1	0.25	0.5	0.75	0.9
Core explanatory variables:					
Internet usage frequency	0.761***	0.595***	0.557***	0.458***	0.379*
	(0.211)	(0.195)	(0.153)	(0.163)	(0.221)
Personal characteristics:					
Age	0.258**	0.232**	0.276***	0.217**	0.257**
	(0.122)	(0.112)	(0.103)	(0.110)	(0.129)
Square of age	-0.003**	-0.002**	-0.002**	-0.002**	-0.003**
	(0.001)	(0.001)	(0.001)	(0.001)	(0.001)
Male	0.698**	0.524*	0.526*	0.593**	0.361
	(0.340)	(0.308)	(0.290)	(0.301)	(0.363)
Urban resident registration	1.207**	1.708***	2.220***	3.019***	3.183***
	(0.543)	(0.503)	(0.395)	(0.421)	(0.645)
Party member	2.280***	0.976***	0.863**	0.810**	0.490
	(0.505)	(0.461)	(0.340)	(0.369)	(0.539)
Characteristics of human capital:					
Health condition	0.526*	0.801***	1.021***	1.238***	1.325***
	(0.270)	(0.250)	(0.196)	(0.209)	(0.320)
Years of education	0.242***	0.313***	0.367***	0.419***	0.464***
	(0.090)	(0.083)	(0.065)	(0.069)	(0.106)
Professional certificate	0.929*	1.113**	1.398***	0.772**	1.048*
	(0.507)	(0.470)	(0.369)	(0.393)	(0.603)
Mandarin level	0.462*	0.720***	0.901***	0.915***	1.034***
	(0.269)	(0.249)	(0.196)	(0.208)	(0.320)
Occupational characteristics:					
Full-time job	1.990**	2.285***	2.310***	2.681***	3.203***
	(0.892)	(0.826)	(0.648)	(0.690)	(1.059)
Within the system	3.297***	3.171***	2.351***	2.330***	2.171***
	(0.541)	(0.501)	(0.393)	(0.419)	(0.643)
Unit scale	0.359**	0.450***	0.687***	0.810***	0.827***
	(0.173)	(0.160)	(0.125)	(0.134)	(0.205)
Family characteristics:					
Family member relationship	0.199*	0.187*	0.202*	0.184*	0.227*
	(0.120)	(0.109)	(0.101)	(0.106)	(0.127)
Family economic situation	0.161	0.197*	0.207*	0.300***	0.330***
	(0.123)	(0.117)	(0.103)	(0.109)	(0.142)
Observations	3,983	3,983	3,983	3,983	3,983

Notes: ①Robust standard error in parentheses.

②*** p<0.01

** p<0.05

* p<0.1.

In addition, as the quantile point rises, the control variables of registered urban residents, health status, years of education, Mandarin level, full-time employment, unit size and household economic status have an increasing impact on employment quality, suggesting that these factors have a greater impact on those in the high employment quality quantile. In contrast, the impact of male, party membership and institutional employment on employment quality tends to decrease as the number of quantile points increasing.

### 4.3 Robustness analysis

Table 5 presents the robustness test results. Considering the possible endogeneity of the model due to omitted variables and bivariate causality, Model 1 shows the regression results of instrumental variables. The instrumental variable is the mean value of the frequency of Internet use in the worker’s community. The effect of internet use on employment quality is still significantly positive and the coefficient value is increased, indicating that the basic findings of this article still hold true after accounting for endogeneity. The model’s minimum eigenvalue of 339.75 is greater than the critical value, suggesting that there is no weak instrumental variable problem. The p-value in the overidentification test is 0.39, so the the instrumental variable is strictly exogenous, and finally the p-value in the Hausman test is less than 0.05, so the frequency of internet use is considered endogenous.

**Table 5:** Robustness analysis results.

	Model 1	Model 2	Model 3	Model 4
	Quality of employment	Quality of employment	Quality of employment	Quality of employment
Internet usage frequency	2.216***		2.832***	2.234***
	(0.477)		(0.726)	(0.487)
Whether use the Internet		1.970**		
		(0.955)		
Control variables	Yes	Yes	Yes	Yes
Observations	3,983	3,983	3,585	3,706

Notes: ① Robust standard error in parentheses.

② *** p<0.01

** p<0.05

* p<0.1.

Model 2 replaces the core explanatory variable with whether or not use the Internet, with those who never use the Internet recorded as 0, who use the Internet recorded as 1. Since the legal retirement age in China is male workers for 60-year-old, female worker for 50-year-old and 55-year-old for female cadres. Some of the preceding samples are outside the legal working age range, which may cause bias in the estimation results, so the sample of employed persons is re-estimated by retaining them according to the legal retirement age. Model 3 shows that the promotional role of Internet use to employment quality remains significant after changing the age range of employed persons. Finally, considering that distrust of the interviewers may affect the authenticity and accuracy of the questionnaire, the sample with low trust in the interviewers is removed and the sample with high trust is retained for re-estimation. Model 4 shows that the promotional role of Internet use to employment quality remains significant after retaining only those respondents with high trust in the interviewer. The results from Model 1-Model 4 show that the findings of this article are robust.

### 4.4 Heterogeneity analysis

Table 6 presents the results of the heterogeneity analysis. In terms of urban-rural heterogeneity, social stratification theory emphasises the division of social groups into different classes with a higher or lower order according to certain criteria, of which urban-rural differences are an important manifestation of social stratification. The urban-rural dichotomy has long existed in China, and on the basis of the urban-rural stratification, the household registration system has a benefit distribution function to the population registration and management function, the urban population occupies a certain advantage in the employment field. The comparison between Model 1 and Model 2 shows that internet use has a positive effect on the quality of employment for both rural and urban workers, but the effect on the quality of employment for urban workers is more pronounced. It can be seen that for every 1 unit increase in the frequency of Internet use, the employment quality of the rural workers increase by 3.108, the urban workers increase by 1.314. This indicates that there are urban-rural differences in the employment quality effect of Internet use.

**Table 6:** The heterogeneity analysis results.

	Model 1	Model 2	Model 3	Model 4
	Quality of employment	Quality of employment	Quality of employment	Quality of employment
	Rural sample	Urban sample	Male sample	Female sample
Internet usage frequency	3.108**	1.314**	1.329**	3.771***
	(0.598)	(0.620)	(0.544)	(0.933)
Control variables	Yes	Yes	Yes	Yes
Observations	2,009	1,974	2,205	1,778

Notes: ① Robust standard error in parentheses.

② *** p<0.01

** p<0.05

* p<0.1.

In terms of gender heterogeneity, gender stratification theory distinguishes men and women into groups with gender hierarchies. The existence of gender stratification makes men and women differ in the field of employment. Therefore the effect of Internet use on the quality of employment for men and women is different. The comparison between Model 3 and Model 4 shows that for every 1 unit of increase in Internet useage frequency, the quality of employment increases by 1.329 and 3.771 for men and women respectively. Internet usage by women at work improves their quality of employment more significantly. A possible explanation for this is that women need to own more internet skills when seeking employment for a higher quality job-seeking, which reaffirms the gender inequality in the labor market.

### 4.5 Mechanism analysis

Table 7 presents an analysis of the mediating mechanisms by which Internet use affects the quality of employment. Model 1 shows that Internet use has a significant positive effect on workers’ employment quality. Model 2 shows that the coefficient of the effect of Internet use on workers’ social capital is 0.224. Model 3 adds both internet use and social capital to the equation for regression and the results show that the coefficient of the effect of social capital on employment quality is 0.045, indicating that social capital improves the quality of employment of workers [45]. The promotional role of internet use on employment quality remains significant after the inclusion of the social capital variable. Thus, social capital plays a partially mediating role in the process of internet use affecting the quality of employment of workers. This confirms the Hypothesis II that internet use not only directly improving the quality of employment for workers, but also indirectly improving the quality of employment for workers through the accumulation of social capital.

**Table 7:** Mediating effect analysis results.

	Model 1 Quality of employment	Model 2 Social Capital	Model 3 Quality of employment
Internet usage frequency	2.216***	0.224**	2.207***
	(0.477)	(0.102)	(0.478)
Social Capita			0.045**
			(0.021)
Control variables	Yes	Yes	Yes
Observations	3,983	3,983	3,983

Notes: ① Robust standard error in parentheses.

② *** p<0.01

** p<0.05

* p<0.1

## 5 Discussion

Based on CLDS data, this article uses factor analysis to construct a system of employment quality indicators, discuss the impact of internet use on workers’ employment quality, and then compare the urban-rural and gender heterogeneity of this impact. Finally analyzes the underlying mechanisms of internet use on employment quality.

First, the measurement of employment quality is relatively complex and is mainly reflected by a combination of social security, subjective satisfaction, job autonomy, job security, career development, salary and compensation, labor hours, and labor relations [24–26]. Most existing studies analyzed the impact of Internet use on employment quality by analyzing one dimension of employment quality, or by analyzing several dimensions separately [31–33], lacking a comprehensive perspective. In this article, we construct a comprehensive index of employment quality and analyze the impact of Internet use on the employment quality index. The results show that Internet use has a significant positive impact on the employment quality of workers.

Internet use is essentially a technological advancement. In terms of the practice of social development, although technological progress may bring about structural unemployment and the widening of the digital divide, technology is intrinsically consistent with the improvement of employment quality, and this intrinsic consistency is mainly reflected in the fact that technological progress itself may have a positive impact on the improvement of employment quality [28]. Furthermore, on the one hand, information technology such as the Internet can build a new employment ecology, mainly in the form of platform, flexibility and borderless employment, which can improve subjective job satisfaction, job autonomy, job security, more flexible labor time and relatively moderate labor relations. On the other hand, the Internet can optimize and upgrade the employment structure. With the popularity of the Internet, economic development has gradually become digitalized, and various industries have been extended by the "Internet+", which has enhanced the activation of the consumer Internet. The Internet is now integrating manufacturing and agriculture, promoting the digital transformation of agriculture and industry through the Internet, realizing digital agriculture and service-oriented manufacturing, giving rise to new industries and new models, creating a large number of new employment methods, thus promoting the continuous optimization of the employment structure, providing workers with better career development and salary compensation. This is conducive to promoting high-quality employment for workers, ultimately achieving free and comprehensive human development.

Second, besides the core explanatory variable internet usage, the quality of workers’ employment is influenced by various other factors. As for individual demographic characteristics, employment quality and age exhibit an inverted U-shaped relationship. As age increases, the quality of employment initially rises and then declines. Male, urban-dwelling, and those affiliated with the Communist Party tend to have higher employment quality, indicating the prevalence of gender, urban-rural, and political affiliation stratification in the employment sector. As for human capital characteristics, individuals with good health, proficiency in Mandarin, and possessing professional certificates tend to have higher employment quality, signifying that investments in human capital can enhance employment quality. As for organizational features, full-time, within-state-owned enterprises, and larger-scale employment settings tend to yield higher employment quality. As for family characteristics, better family relationships and improved family economic status correlate with higher employment quality. It shows that the influencing factors on employment quality are multidimensional. Apart from the core explanatory variable of internet usage in this article, a comprehensive analysis from an all-round perspective is also needed to consider other influencing factors.

Third, previous studies paid less attention to the heterogeneity across workers when analyzing the impact of Internet use on employment quality [20, 27]. This article pays attention to this heterogeneity and finds that the regression coefficient tends to decrease as the employment quality quantile continues to increase. Internet use has a greater impact on workers in the lower employment quality quantile, indicating that there is diminishing marginal utility in the positive effect of Internet use on employment quality. Compared to workers with higher employment quality, those with low employment quality are less competitive for jobs, and the effects of Internet use on this group will be more sensitive. The effect of Internet use on workers’ employment quality is heterogeneous, with stronger effects on rural workers and female workers. This result suggests that urban-rural inequality and gender inequality do exist in the Chinese labor market, and that the root of inequality is still in the system [41].

Finally, few studies have analyzed the internal mechanisms by which Internet use affects the impact of employment quality [31]. By analyzing the indirect role of social capital in the process of the Internet’s impact on employment quality, this article aims to make the research more in-depth. Social capital is both a prerequisite for mutual collaboration among members and a resource that drives the strengthening of social networks, an institutionalised network of relationships [36]. Social capital is not possessed by individuals and cannot be acquired without effort, but needs to be acquired from social networks through practice, and social resources play a more important role than individual resources in the later stages [37]. The use of the Internet has a positive effect on the level of individual social capital, and with the increased accessibility of information, social resources are better integrated. The Internet provides a platform for people to communicate and share information with each other, broadening social networks and contributing to the accumulation of social capital [39]. In addition, some studies have analyzed the positive impact of social capital on employment quality, viewing social capital as an individual’s ability to mobilise and draw resources from a wide range of networks, with more resources meaning more opportunities for high-quality employment, and thus social capital can improve the quality of employment for workers [40]. However, related studies have neglected the indirect role of social capital in the impact of Internet use on employment quality. The results of this article show that social capital is an intrinsic mechanism through which Internet use affects the quality of employment, and that while Internet use directly improves the quality of employment of workers, it also indirectly improves the quality of employment of workers through the accumulation of social capital (S1 Appendix).

## 6 Implications, limitations and future recommendations

### 6.1 Implications

The policy implications of this article are: First, Internet use is conducive to the improvement of the quality of employment for workers, especially those with low quality levels of employment. The current level of Internet infrastructure and Internet penetration in China needs to be further improved, the government should increase investment in Internet infrastructure to ensure a comprehensive and effective supply of information networks.

Second, the existence of the household registration system and gender discrimination are at the root of the differences in the impact of Internet use on the quality of employment of workers, efforts should be made to promote information technology in rural areas and to address the imbalance between urban and rural Internet development. Meanwhile, a unified and open labor market should be established to eliminate gender differences in employment.

Third, the accumulation of human capital helps to improve the quality of employment. The strategy of strengthening the country through science and education should be further promoted, and professional skills training should be strengthened while education is emphasized. Along with the improvement of workers’ own education level, they can better adapt to the needs of the digital economy, use the Internet to find employment opportunities that better match their own characteristics and meet the demand for fuller and higher quality employment.

Fourth, social capital is an intrinsic mechanism through which the use of the Internet affects the quality of employment. Online platforms should be actively nurtured to create conditions that broaden the channels through which people can accumulate social capital. The supervision of online platforms should be stepped up to stop the spread of false and fraudulent information, more safe, efficient and high-quality employment information sharing platforms should be cultivated and built.

### 6.2 Limitations and future recommendations

The limitations of this article are: First, as some of the key variable indicators in the CLDS vary in each period of data and there are differences in the statistical calibre, this article uses cross-sectional data in analyzing the impact of internet use on employment quality, which lacks sufficient time series. This also results in the relative lack of innovativeness in the model of this article. Second, in this article, employment quality is measured more comprehensively based on a number of dimensions such as social security, subjective satisfaction, job security, job autonomy, career development, salary, working hours, and labor relations, but a unified measure of employment quality has not yet been formed in the academic community, a more comprehensive and authoritative indicator system needs to rely on more data to be achieved. The above limitations are also directions that will continue to be explored in the future.

## Supporting information

S1 Appendix (DOCX)
