# Supplementary figures and images for: Does internet use improve employment?——Empirical evidence from China
Source: PLoS One. 2024 Apr 16;19(4):e0301465. doi: 10.1371/journal.pone.0301465 (PMC11020373; doi:10.1371/journal.pone.0301465)

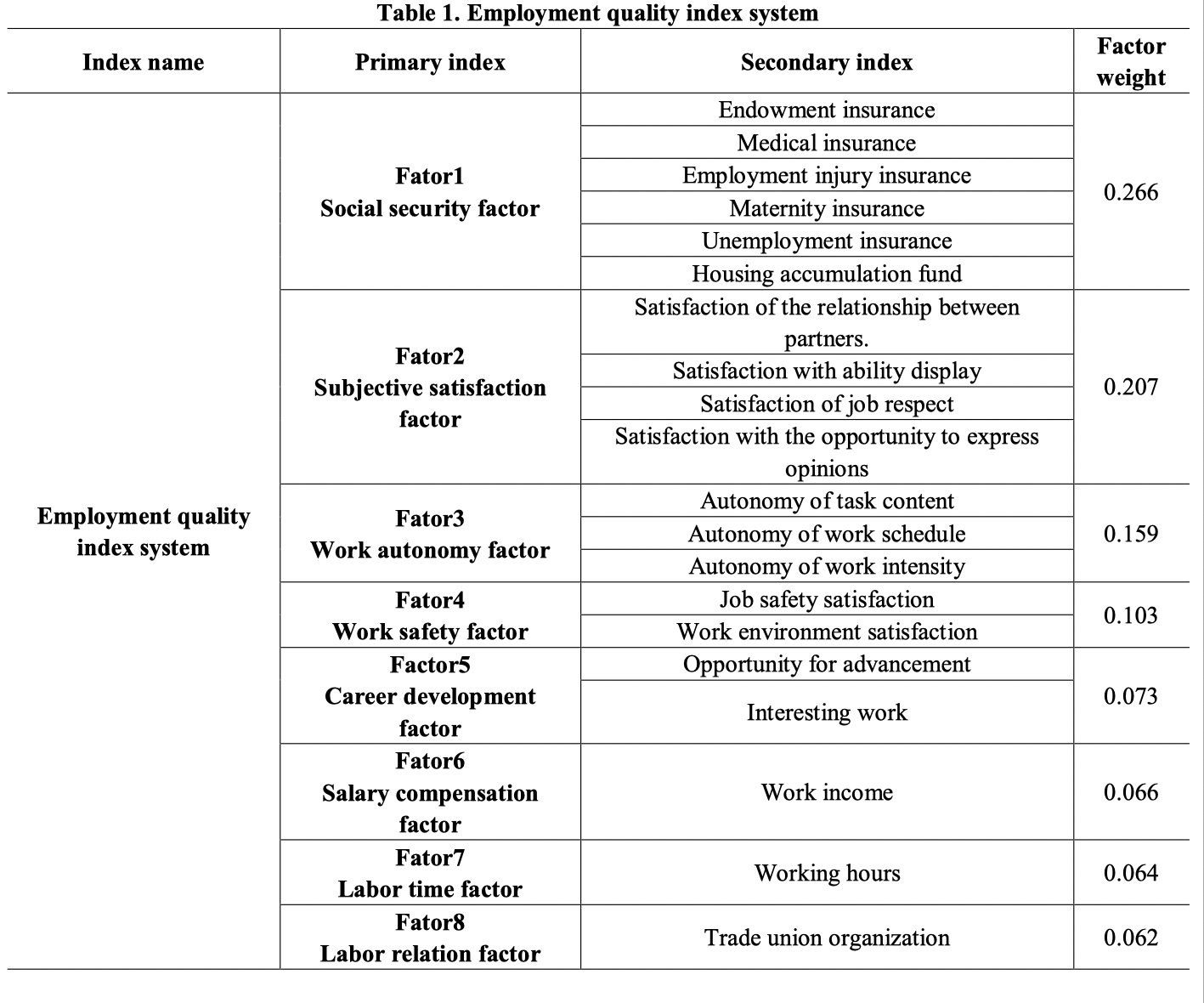

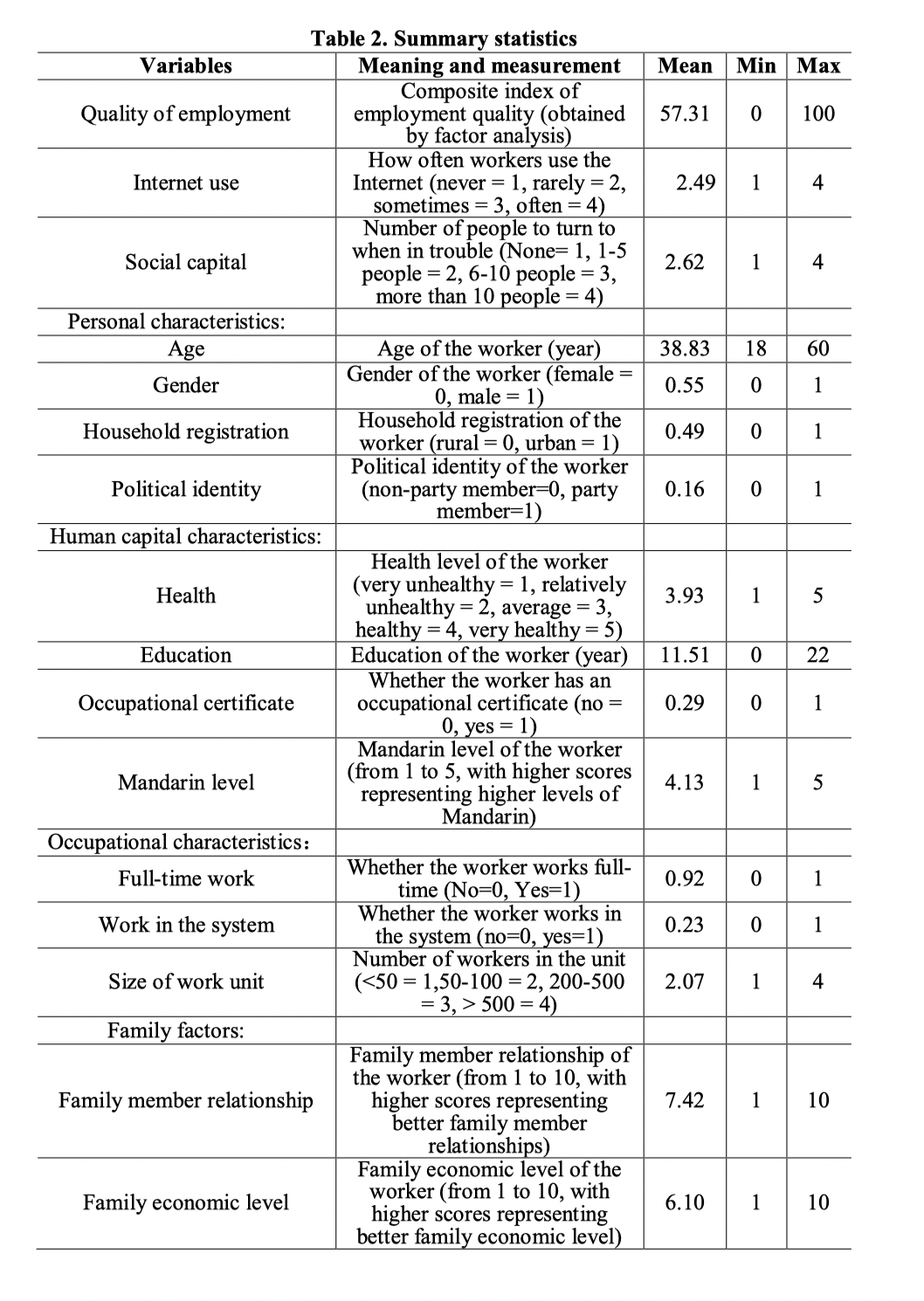

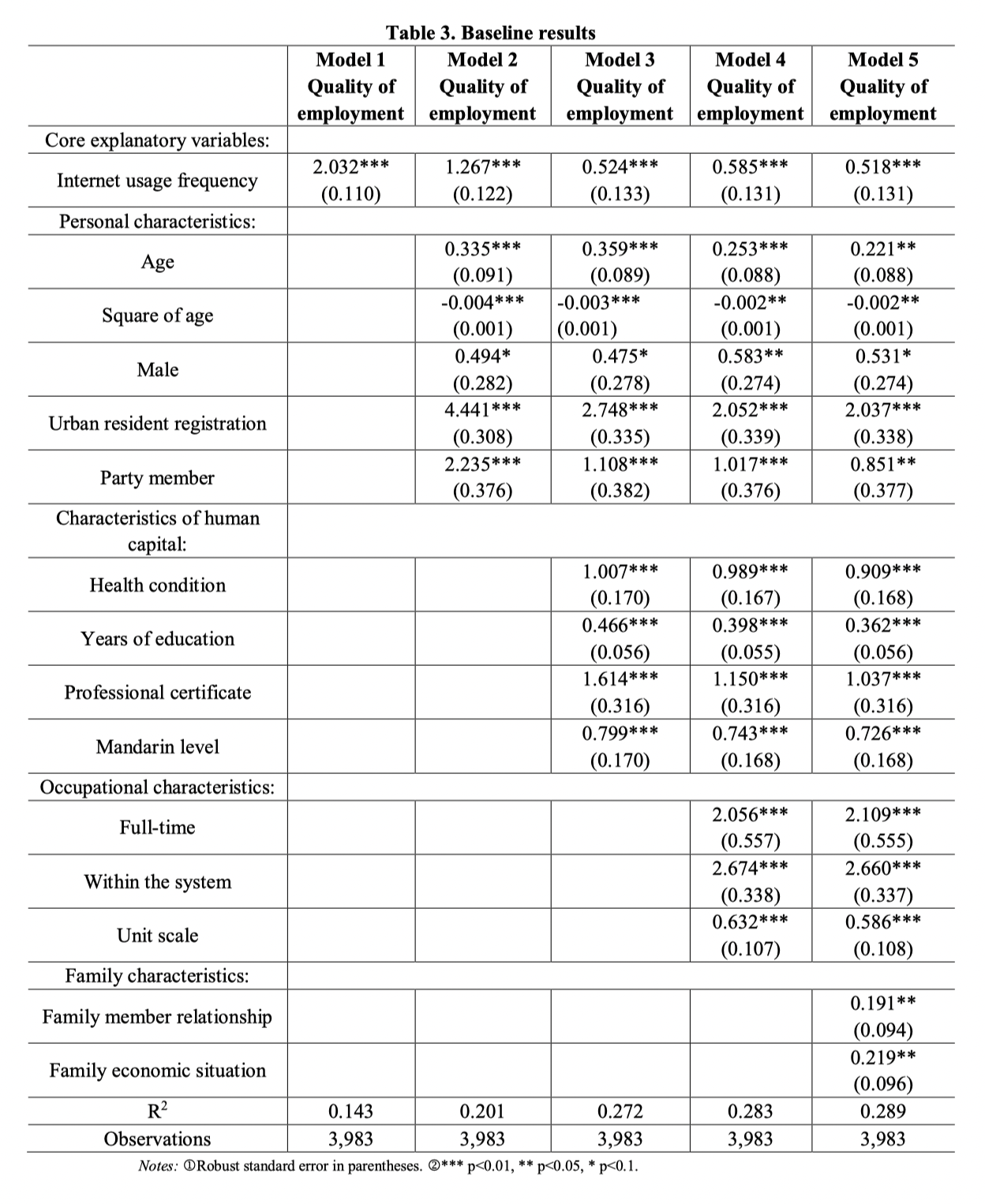

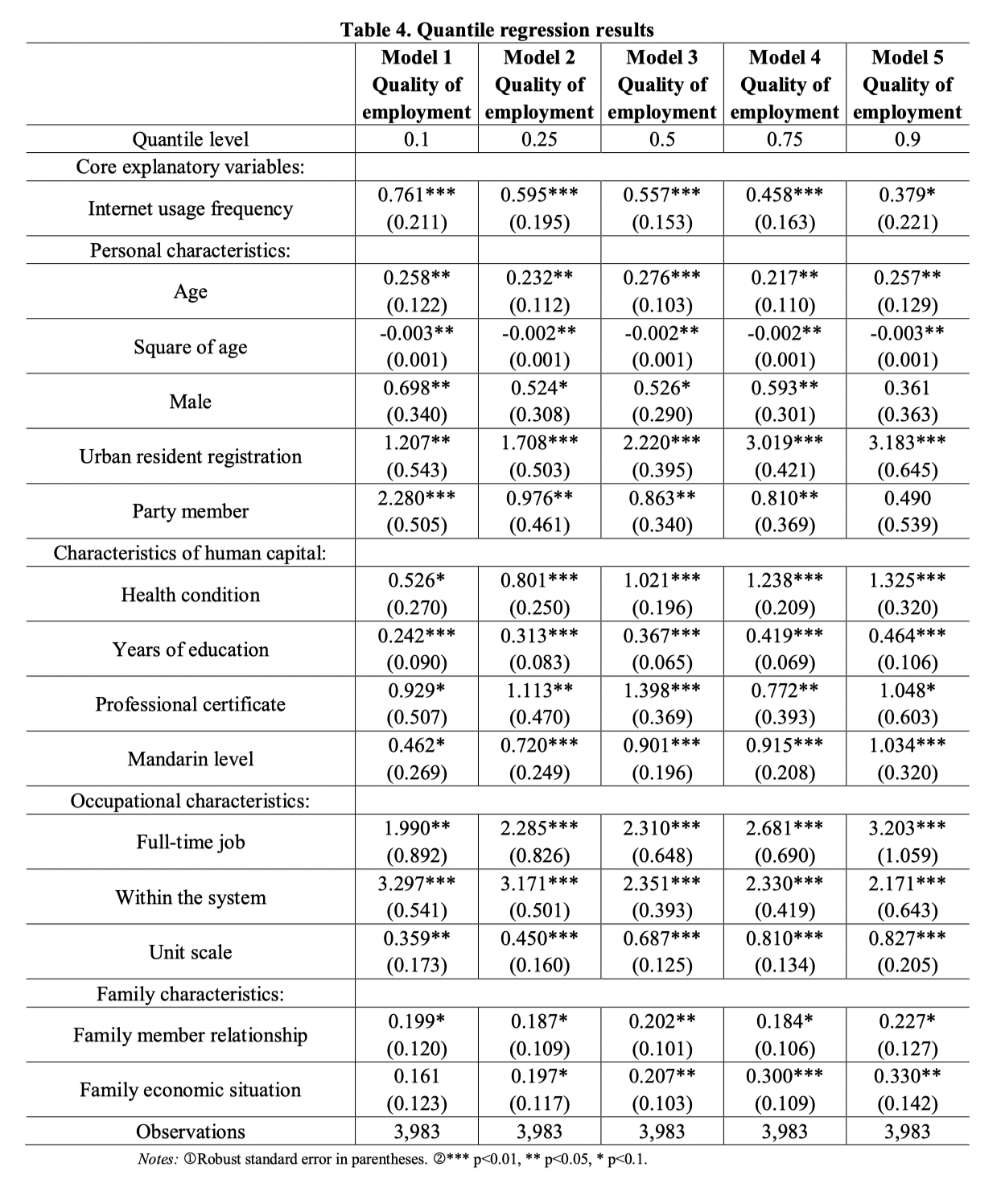

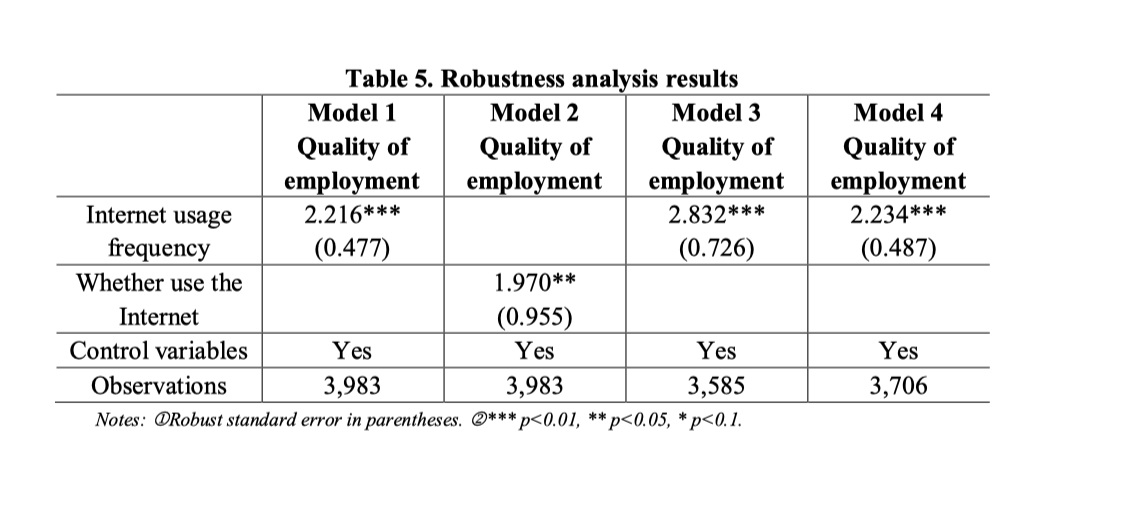

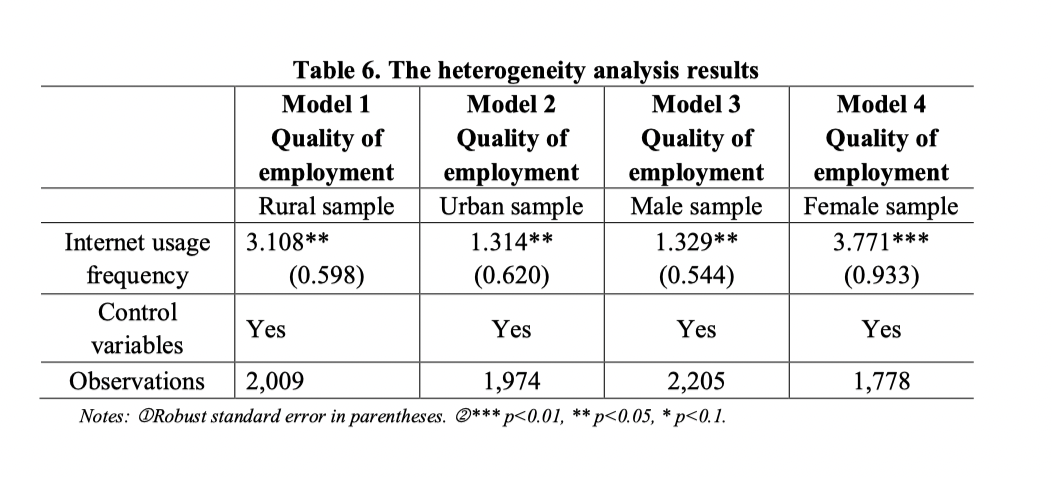

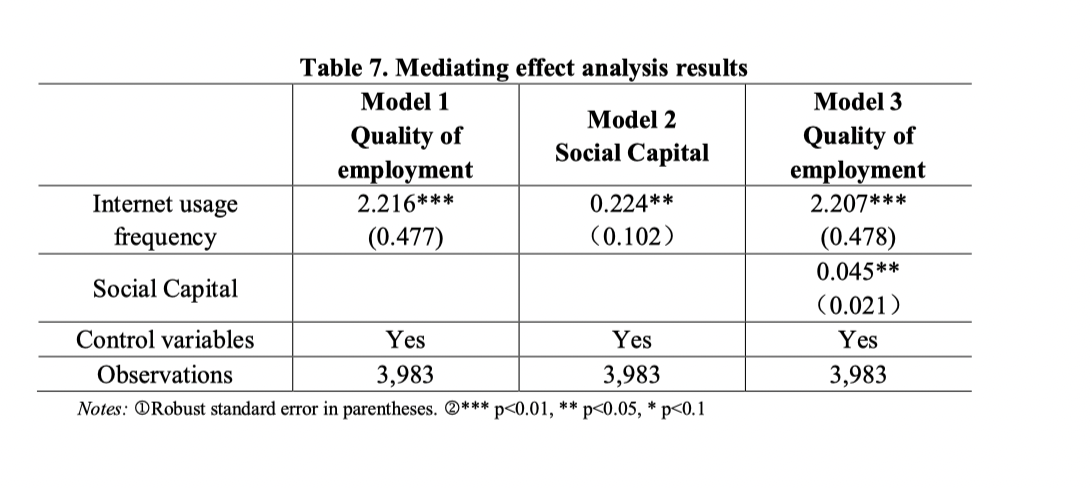

Supplement: S1 Appendix — (DOCX) [file pone.0301465.s001.docx]
